# Cost-effectiveness of stimulation of the sphenopalatine ganglion (SPG) for the treatment of chronic cluster headache: a model-based analysis based on the Pathway CH-1 study

**DOI:** 10.1186/s10194-015-0530-8

**Published:** 2015-05-21

**Authors:** Jan B. Pietzsch, Abigail Garner, Charly Gaul, Arne May

**Affiliations:** Wing Tech Inc., Menlo Park, CA USA; Migräne- und Kopfschmerzklinik Königstein, Königstein im Taunus, Germany; Institut für Systemische Neurowissenschaften, Universitätsklinikum Hamburg-Eppendorf, Martinistr. 52, Haus S10, Zi. 318, Hamburg, 20246 Germany

**Keywords:** Chronic cluster headache, Implantable stimulator, Stimulation, Sphenopalatine ganglion, Cost-effectiveness, Germany

## Abstract

**Background:**

In the recent Pathway CH-1 study, on-demand stimulation of the sphenopalatine ganglion (SPG) by means of an implantable neurostimulation system was proven to be a safe and effective therapy for the treatment of chronic cluster headache. Our objective was to assess the cost-effectiveness of SPG stimulation in the German healthcare system when compared to medical management.

**Methods:**

Clinical data from the Pathway CH-1 study were used as input for a model-based projection of the cost-effectiveness of SPG stimulation through 5 years. Medical management as the comparator treatment was modeled on the basis of clinical events observed during the baseline period of CH-1. The costs of treatment were derived from a previously published cluster headache costing study and 2014 medication, neurostimulator, and procedure costs. We computed the 5-year incremental cost-effectiveness ratio (ICER) in euros per quality-adjusted life year (QALY), with costs and effects discounted at 3 % per year.

**Results:**

SPG stimulation was projected to add 0.325 QALYs over the study period, while adding €889 in cost, resulting in a 5-year ICER of €2,736 per QALY gained. Longer follow-up periods, higher baseline attack frequency, and higher utilization of attack-aborting medications led to overall cost savings. SPG stimulation was found either cost-effective or cost-saving across all scenarios investigated in sensitivity analyses.

**Conclusions:**

Our model-based analysis suggests that SPG stimulation for the treatment of chronic cluster headache, under the assumption of sustained therapy effectiveness, leads to meaningful gains in health-related quality of life and is a cost-effective treatment strategy in the German healthcare system.

## Background

Treatment of chronic cluster headache (cCH) is guided by the dual objectives of ending acute attacks and decreasing attack frequency. Current medical treatment for acute attacks includes the use of oxygen inhalation, subcutaneous or intranasal application of triptans, or intranasal lidocaine [1–3]. Preventative medications usually consist of verapamil or lithium as first choice; alternatively or concurrently, steroids, topiramate, melatonin, long-acting triptans, and occipital nerve blocks are also commonly used as prophylactic treatments [4–6]. The costs of such pharmaceutical treatments for cCH are substantial, and have previously been estimated to average €15,700 per year, with severely afflicted patients incurring even higher annual medication costs [7].

Electrical stimulation of the sphenopalatine ganglion (SPG) has recently been proposed as an alternative treatment approach for cluster headache, relying on an implantable on-demand stimulator that is activated by the patients themselves (PULSANTE SPG Microstimulation Therapy; Autonomic Technologies Inc. (ATI); Redwood City, California, USA). The safety and effectiveness of the PULSANTE system was first investigated in the Pathway CH-1 study, a multicenter, randomized trial [8]. This study found the therapy to have dual clinical benefits of acute pain relief and attack prevention, while demonstrating an acceptable safety profile comparable to similar surgical procedures. In recent years, further investigational and commercial experience with the system has been gained in the European Union and other markets [9, 10]. In the EU, the PULSANTE system is available for the treatment of episodic and chronic cluster headache since obtaining CE mark approval in 2012. In the United States, a pivotal study is currently recruiting patients (NCT02168764).

Our objective in this study was to evaluate the long-term cost-effectiveness of SPG stimulation, as compared to medical management, based on the findings of the Pathway CH-1 study and a model-based extrapolation through 5 years. We chose the German healthcare system as the setting for this analysis because of the experience already gained with SPG stimulation in that market, and the general relevance of Germany as the largest European healthcare system.

## Methods

### Study design

Our analysis relied on two primary sources of data. First, we used data from the Pathway CH-1 study to estimate the therapeutic effectiveness of SPG stimulation compared to medical management. Second, we used data from a comprehensive resource utilization and costing study previously conducted on CH patients in the German healthcare system [7] to estimate current costs of cCH management in the acute phase.

For conventional medical management, resource use and health-related quality of life were estimated according to the baseline values of the Pathway CH-1 study, prior to implantation of the SPG neurostimulator [8]. These values were assumed to remain constant throughout the period of the modeled analysis. In line with the results of the long-term data available to-date [9, 10], long-term effectiveness of SPG stimulation for accomplishing pain relief was assumed to remain constant over time, and to be identical to the effectiveness observed in the Pathway CH-1 study.

For each treatment strategy, we computed the total costs as well as gains in health-related quality of life, as measured through projected gains in quality-adjusted life years (QALYs). The timeframe of the base case analysis was 5 years. We chose this comparatively short timeframe in order to balance our objective of performing a conservative analysis (in light of the fact that follow-up data are currently available for only 3 years), against the typical requirement that model-based assessments of therapies for chronic conditions employ a long-term or even lifetime perspective.

### Model structure and modeling framework

The study model computed costs and QALYs for two simulated cohorts: cCH patients treated with SPG stimulation, and cCH patients treated with medical management (referred to as the control cohort). We assumed that all cCH attacks not successfully treated with SPG stimulation (i.e., attacks in which pain relief was not achieved within 15 min, in line with the CH-1 primary efficacy endpoint) would instead be treated with standard medical management.

The model further assumed that stimulation would reduce the average frequency of cCH attacks in the SPG-treated cohort by 31 %, as was observed in the Pathway CH-1 study [8]. When considering the overall cohort of frequency responders and non-responders, we conservatively assumed that this prophylactic effect would gradually decline by 10 % each year, so that the prophylactic effect would be reduced by half at 5 years of follow-up.

**Fig. 1:**
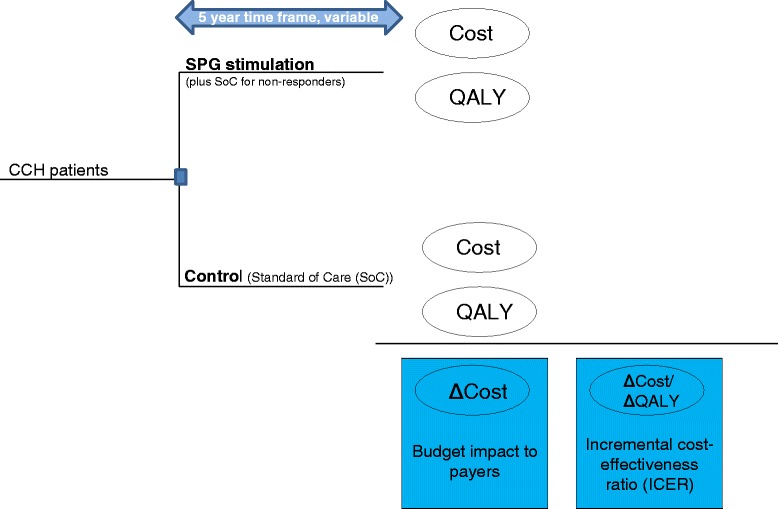
Representation of model structure

Our analysis had two co-primary outcome measures: the per-patient budget impact to payers; and the incremental cost-effectiveness ratio (ICER), defined as the incremental direct costs of medical treatment and consequences divided by the incremental health benefits expressed as quality-adjusted life years (QALYs). The ICER is a common metric used in health-economic analyses to assess the value of an intervention [11]. QALYs, taking into account both gains in survival and health-related quality of life, describe the direct patient-relevant benefits associated with a therapy or intervention [12]. Figure 1 provides an overview of the model structure and resulting outcome metrics.

The analysis took the perspective of payers in the German statutory health insurance system. Costs and health outcomes were discounted at 3 % per year, in line with current guidelines for health-economic analysis [13]. All costs were actual or estimated amounts in 2014 euros. Mortality was not considered in the analysis because of the comparatively young treatment age of patients in Pathway CH-1, the limited 5-year timeframe of the analysis, and because no significant differences in mortality were expected between the SPG stimulation and medical management cohorts.

#### Input parameters

The Pathway CH-1 study and its findings have been presented elsewhere [8]. In short, that study was a prospective, randomized, blinded, multicenter trial conducted to evaluate the safety and efficacy of acute electrical stimulation of the SPG using the PULSANTE SPG Microstimulation Therapy (formerly referred to as ATI Neurostimulation System). Of note, this study exclusively recruited patients with chronic cluster headache. While inclusion criteria did not require cCH to be medically refractory, most of the ultimately enrolled patients were refractory. The study did not collect cost data.

For each study subject (for demographic data see Table 1), the Pathway CH-1 trial consisted of 5 phases [8]: 1) a pre-implant period of 4 weeks to allow for establishment of subjects’ baseline attack frequency; 2) a post-implant period of at least 3 weeks for recovery after implantation; 3) a therapy titration phase of at least 6 weeks, during which the stimulator was used, and the selected electrodes and stimulation parameters were individually adjusted for optimal therapy; 4) an experimental phase lasting a minimum of 3 weeks and a maximum of 8 weeks, or the shortest period required for the treatment of 30 attacks; and 5) an open-label phase to continue collecting data through 1 year after implantation, with patients receiving full stimulation therapy at each use.

**Table 1:** Baseline characteristics of patients enrolled in the Pathway CH-1 study [8]

Variable	Pathway CH-1 (*N* = 28)
Age (years)	45 (20–63)
Male gender (no.; %)	27 (84 %)
Baseline CH attacks per day	2.74 (0.57–10)

The primary endpoint for this sham-controlled study for gauging effectiveness was pain relief in the acute attack within 15 min after starting the stimulation, and without use of rescue medications, as defined in the study protocol. A secondary observational endpoint was the reduction of attack frequency, established by comparing the frequency of cCH attacks per 4 weeks at the end of the experimental period to the frequency established at baseline. Health-related quality of life was assessed using the physical (PCS) and mental (MCS) component summary scores of SF-36v2. A full list of all endpoints is provided in the published Pathway CH-1 study [8].

##### Therapy effectiveness

Our model assumed an attack frequency for the stimulation cohort that was 31 % lower than the baseline frequency of 2.74 attacks per day. This estimate was based on the study-reported percentage of frequency reduction across the entire SPG-treated cohort (i.e., frequency responders and non-frequency-responders). The medical management cohort was assumed to maintain its baseline attack frequency. Over the 5-year modeling horizon, the attack frequency of the medical management cohort was assumed to remain constant, while the prophylactic effect in the SPG-treated cohort was assumed to gradually decline over time (see Table 2).

**Table 2:** Model input parameters

Variable	Base case	Range	Source
**Cohort characteristics**			
Age	45		Pathway CH-1 data [8]
Male gender	84 %		
**Clinical effectiveness**	**Base case**	**Range (%)**	**Source**
Baseline CH attacks per day	2.74	±50	Pathway CH-1 data [8]
Pain relief with SPG stimulation (within 15 min)	67.1 %	50.2–80.5	
Frequency reduction with SPG stimulation (total cohort)	31.0 %	15–50 (assumption)	
Absolute decline in frequency reduction for SPG stimulation, per year	3.1 %	0–6.2	Author estimate
**Medication costs** ^**a**^	Base case (€)	Range (%)	Source
Oxygen [2.6 * inhalation]	257.20		Utilization data based on [7], frequency-adjusted to Pathway CH-1 cohort; 2014 unit cost data from [14]
Zolmitriptan nasal spray [1.08 * 5 mg]	1,118.08		
Sumatriptan s.c. [1.30 * 6 mg]	2,959.64		
Sumatriptan nasal spray [1.08 * 20 mg]	112.62		
Resulting mean medication cost per attack	8.92	±25	
**SPG stimulation costs**	**Base case (€)**	**Range**	**Source**
Implantation of SPG stimulation system (hospital inpatient)	5,293.99		DRG B17-B, based on ICD-10-GM diagnosis code G.44 and OPS procedure code 5–059.c2
Reimbursed cost of ATI SPG Neurostimulator	25,000.00		Estimate provided by manufacturer
CT/CVT imaging cost pre- and post-implant	400.00		Author estimate
6 visits to headache center for device titration, follow-up to implantation	596.46		Based on cost reported in [7], adjusted to 2014
Revision of implant (4 of 32 patients)	5,293.99		DRG B17-B, based on ICD-10-GM diagnosis code G.44 and OPS procedure code 5–059.c2; implant provided by manufacturer (ATI) at no additional cost [26].
Antibiotics for infection treatment (3 of 32 patients)	94.88		Augmentan (amoxicillin) tablets N2 [14]
Device explantation, without new implantation (2 of 32 patients, outpatient)	355.77		Ambulatory surgery reimbursement, EBM 31,251, 31,504, 31,670, based on OPC code 5–028.6 [27]
**Utilities** ^**b**^	**Base case**	**Range**	**Source**
Baseline	0.55		Approximation based on Pathway CH-1 SF-36v2 data and mapping algorithm [15]; years 2–5 extrapolated
End of experimental phase (stimulation)	0.67		
Open label (stimulation)	0.68		
12 months (stimulation)	0.61		
Years 2–5 estimate (stimulation)	0.61		

a. 6 months, average total costs per patient, medical management cohort; [mean daily intake in patients taking respective medication]

b. mapped EQ-5D scores, based on trial-reported SF-36

Based on the Pathway CH-1 study protocol, we further assumed that for attacks that were successfully treated with stimulation (i.e., pain relief was accomplished within 15 min of onset), no acute rescue medication was required – in line with the definition of this endpoint, which was defined to only be met if no acute medication was taken by the patient. Conversely, it was assumed that those attacks not successfully treated through SPG stimulation did require acute rescue medications at the average doses, as described below.

In terms of therapy complications, our model takes into account device revisions that needed to be performed in CH-1. No longer-term revisions are assumed to be required, because the SPG stimulation system does not have a battery that needs to be replaced, a difference to other stimulation technologies such as spinal cord stimulation (SCS) and occipital nerve stimulation (ONS). Further, our analysis takes into account two device explants and oral antibiotic treatment of surgical site infections in two patients. Other adverse events listed in the CH-1 study either did not require treatment or did not lead to additional treatment costs, and are therefore not considered in this analysis.

##### Costs

Costs were considered from the perspective of German statutory health insurance (SHI), taking into account only direct healthcare costs. The cost of implanting the SPG stimulation system was based on the 2014 G-DRG reimbursement amount defined by the ICD-10-GM diagnosis code for cluster headache, and the applicable procedure code for implantation of the neurostimulator. In addition to the DRG payment, the cost of the stimulation system itself was also taken into account, in accord with the amounts currently paid to hospitals by insurance payers. Further, we estimated the costs of pre- and post-implant imaging by computed tomography (CT) and digital volume tomography (DVT), as well as costs for revisions, explants, and antibiotic treatment of 3 patients suffering from surgical site infection. Also, we included the costs of 6 follow-up visits to an outpatient headache center for device titration and general follow-up of the implantation procedure. Beyond these 6 visits, we assumed no differences between costs of physician visits between the stimulation and the control cohorts, although patients with effective therapy will not seek medical advice or care as often as intractable patients. Of note, all patients in the CH-1 study were medically intractable.

Total costs for attack-aborting medications were computed on the basis of the utilization rates reported for chronic cluster headache patients in the prior costing study [7] and 2014 medication costs [14], following the same approach as that taken in the original study. For the medical management cohort of our model, the resulting cost for attack-aborting medication was €8,892 per year (€171 per week). This value reflects the lower attack frequency of 2.74 attacks per day in the Pathway CH-1 study [8], compared to 3.80 in the costing study [7]. We did not consider costs for preventative medications, as these do not differ between the two cohorts, and therefore do not influence the results of our incremental analysis.

##### Health-related quality of life

Utility estimates were derived as follows. Short-Form 36v2 (SF-36) health profile data was collected alongside the Pathway CH-1 study. We used an established mapping algorithm [15] to convert the mean statistics of the 8 dimension scores of the SF-36 into mean cohort EQ-5D preference-based index scores. The SF-36v2 forms were available to CH-1 study participants at baseline, at the end of the experimental period, and during the open-label period, as well as during follow-up at 12, 15, 18, 21, and 24 months.

For the simulated medical treatment cohort, we assumed the baseline utility would be maintained over the full timeframe of the analysis. For the stimulation cohort, we determined utility projections in 4-week increments based on the mapped EQ-5D scores at the reported time points up to 24 months. For the period from 3 to 5 years, we assumed quality of life to be constant at the average of the year-projected scores between 12 and 24 months, thus extrapolating the average quality of life observed in year 2 to years 3, 4, and 5.

The utility values were subsequently used in the analysis to compute quality-adjusted life years (QALYs) for both the medical management and stimulation cohorts.

#### Analysis of uncertainty

Comprehensive one-way sensitivity analyses were conducted to evaluate the effects of parameter uncertainty, including variations in the effectiveness of SPG stimulation. The parameter ranges were derived from the published Pathway CH-1 results, prior published data, and from expert opinion where applicable (see Table 2).

## Results and discussion

### Base case results

Over the 5-year time horizon of the analysis, discounted total direct medical costs for the SPG stimulation cohort were €42,187, compared to €41,298 for the simulated medical management cohort, for a total additional cost of €889.

Health-related quality of life for the stimulation cohort, estimated on the basis of subjects’ SF-36 scores mapped to EQ-5D preference scores, was 0.548 at baseline, 0.668 at the end of the experimental period, 0.675 during the open-label period, and 0.614 at 12 months. The scores for year 2, based on collected SF-36 information, were 0.606 at 15 months (based on *N* = 25), 0.602 at 18 months (*N* = 23), 0.592 at 21 months (*N* = 17), and 0.683 at 24 months (*N* = 15).

Using the described extrapolation of quality-of-life scores, and assuming that quality of life for the medical management cohort would remain stable at the baseline level, the undiscounted QALY gain for the SPG stimulation cohort was 0.086 in year 1, and 0.066 in each following year through year 5. The resulting discounted QALY gain over 5 years was 0.325.

As a result of the overall savings and projected QALY gains associated with stimulation the incremental cost-effectiveness ratio (ICER) at 5 years was €2,736 per QALY gained (see Table 3).

**Table 3:** Health outcomes and incremental cost-effectiveness results at 5 years

	Costs (€)			Effects (QALYs)			ICER (€/QALY)
	SPG	Control	Difference	SPG	Control	Difference	
Base case, 5 years, discounted	42,187	41,298	+889	2.87	2.55	0.32	2,736
Base case, 5 years, undiscounted	42,998	44,475	−1,477	3.09	2.74	0.35	<0; SPG dominating

### Uncertainty analyses

Table 4 provides an overview of the most relevant scenario results, including the discounted ICERs and discounted cost differences for SPG stimulation versus medical management at 5 years. The cost-effectiveness projections were found to be robust across a wide range of assumptions, including variations in pharmaceutical costs, attack frequency, and the assumed percentage of frequency responders among stimulation patients.

**Table 4:** Sensitivity analyses: key scenarios and corresponding ICERs and absolute cost differences for SPG versus medical management cohorts

Scenario	ICER	Absolute cost difference, SPG vs. control (€)
	(€/QALY)	
Base case	2,736	+889
Base case (undiscounted)	SPG dominating	−1,477
Pain relief 50.2 % (lower bound of 95 % CI)	18,846	6,125
Pain relief 80.5 % (upper bound of 95 % CI)	SPG dominating	−3,262
1.37 CH attacks per day (50 % of baseline)	50,590	16,442
4.11 CH attacks per day (150 % of baseline)	SPG dominating	−14,664
Utilization (cost) of attack-ending meds +25 %	SPG dominating	−6,887
Utilization (cost) of attack-ending meds −25 %	€26,663	8,665
No frequency response considered for SPG	13,180	4,284
Average frequency reduction low (15 %)	8,126	2,641
Average frequency reduction high (50 %)	SPG dominating	−1,191
No annual change in frequency response	220	72
Absolute annual reduction in frequency response of 6.2 % (double of base case assumption of 3.1 %)	5,251	1,707
Sumatriptan s.c. used by every patient (at dosage reported by [7], frequency-adjusted to Pathway CH-1; total medication cost per attack: €18.81)	SPG dominating	−33,609
Every attack treated by maximum guideline-defined medication dosages (total medication cost per attack: €56.66)	SPG dominating	−165,618
Assumption that stimulation device in required revisions would be paid by payers, as opposed to the manufacturer	12,351	4,014
Reduced timeframe of analysis 3.5 years	40,058	9,537
Extended timeframe of analysis 7.0 years	SPG dominating	−9,857

Unless otherwise noted, discounting of 3 % is applied on all costs and effects

**Fig. 2:**
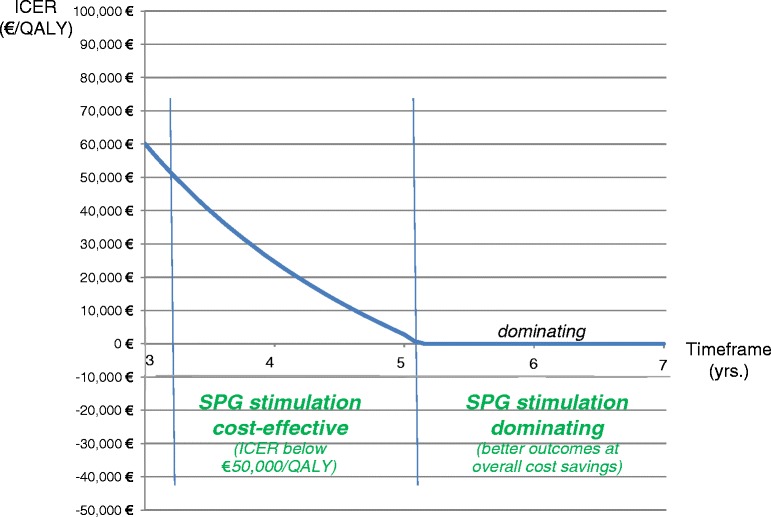
Cost-effectiveness results over time horizons of 3–7 years. Note: Considered willingness-to-pay threshold €50,000/QALY, based on [25]. Per common convention, no negative ICERs shown for range where SPG is dominating

Reducing the timeframe of the analysis to a follow-up period of 3.5 years resulted in an ICER of €40,058, at an added discounted cost of €9,537. When the timeframe of the study was increased to 7.0 years, SPG was dominating, at a total discounted savings of €9,857. Figure 2 provides an overview of the ICER results for the various timeframes considered.

## Discussion

While cluster headache patients have benefitted from some meaningful advances in drug therapy since the 1990s, when injectable and intranasal triptans were introduced, no substantial improvements in pharmaceutical therapy have been made since. As a result, a sizable number of cCH attacks remain insufficiently treated, and drug therapy alone has been unable to achieve pain relief sufficiently. At the same time, currently used medications, both abortive and prophylactic, are associated with a number of potential severe side effects and tolerability concerns, including increased risk of cardiac events [16, 17] and hypertension [18].

Furthermore, [16–20] injectable triptans are frequently associated with “triptan sensations,” characterized by distressing chest discomfort, palpitations, and flushing. Patients with more frequent cluster attacks per day are unable to use triptans because of the dose limit they can use in a 24 h period. In addition, triptan treatment is contraindicated for patients with cardiovascular disease, and around 10 % to 20 % of patients are not effectively treated by—or are resistant to—these therapies. High doses of preventive medications like verapamil have the potential to cause serious cardiac abnormalities, including bradycardia, heart blocks and severe hypotension needing aggressive and expensive interventions. Also, lithium is frequently not well tolerated and requires careful medical management of the patients. Topiramate is increasingly used but due to central side effects is not well tolerated by a substantial number of patients. Some patients respond only to corticosteroids, or need them frequently because other preventative medications are not effective throughout the therapy; in these patients severe side effects are unavoidable. Methysergide and injectable dihydroergotramine are rather effective in cluster headache therapy but currently unavailiable in most countries.

As a result, there is a continued need for improved or complementary treatment approaches for cCH patients. This pressing clinical need is further underscored by a recent study reporting the burden of cCH to patients and society: more than 50 % of cCH patients reported suffering symptoms of depression; 25 % were found to have suicidal tendencies; and about 25 % received some form of disability allowance [21].

SPG stimulation using a permanent implantable neurostimulation system has recently been demonstrated to be a safe and effective treatment alternative that offers acute pain relief at effectiveness levels comparable to the latest drug therapies, while also having the beneficial effect of reducing the frequency of attacks [8, 10]. To-date, it is the only therapy that has the potential of dual benefits of acute pain relief and a reduction in attack frequency, combined with a reduction in overall medication use [10].

The costs of current drug treatments for cCH are staggering, averaging more than €12,000 per patient per year for acute medications alone. SPG stimulation has the potential to lower these costs, by reducing or eliminating the need for attack-ending medications, and by achieving preventive effects [8–10].

At the same time, implantation of an SPG stimulation system is a significant one-time investment for payers upfront, averaging more than €30,000 for implantation, when the costs of the device and the implantation procedure are considered. Understanding the health-economic profiles of a drug-based treatment strategy versus an implantable neurostimulation strategy is therefore essential to inform decision-making by payers and providers.

Despite the upfront costs for the device and implantation, our analysis showed that SPG stimulation has the potential to be cost-effective in time frames as short as 3 years, and to reduce overall costs to payers in timeframes greater than 5 years, primarily through the reduced need for rescue medications, but also by means of its observed preventive effects.

The results of this study compare favorably to the cost-effectiveness findings for a number of well-accepted and currently reimbursed implantable neurostimulation systems used to treat other clinical conditions with high disease and cost burden, including deep brain stimulation for movement disorders [22], spinal cord stimulation for failed back syndrome [23], and cochlear implants [24]. These comparable therapies have been found to be cost-effective, in that their increased upfront costs have been shown to be associated with improved outcomes that healthcare systems considered a worthy investment.

But none of those technologies were found to bring about cost savings at time horizons as short as those projected in our analysis of SPG stimulation. While the assessment methods used in these published studies resemble our own—in that the results were derived from model-based projections informed by shorter-term clinical trial results—the timeframes adopted for those analyses were a minimum of 5 years, and sometimes a lifetime horizon. The timeframe of 5 years used in our analysis is therefore conservative. Longer follow-up would have led to an even more favorable health-economic profile. This fact is worth noting in light of the relatively young age of cCH patients, as evidenced by the average age of 45 years for the subjects included in Pathway CH-1.

The results of our sensitivity analyses show effects on the cost-effectiveness profile of SPG stimulation that—directionally—would be expected. In the control cohort, higher baseline attack frequency leads to additional savings because more attacks need to be treated with medications. In the SPG stimulation cohort, higher SPG therapy effectiveness leads to overall cost savings at follow-up longer than 5 years, while lower therapy effectiveness reduces the cost-effectiveness, as evidenced by an increased ICER. However, it is noteworthy that SPG stimulation remained cost-effective or cost saving across all trial-informed scenarios, even when considering the lower bound of the 95 % confidence interval for therapy effectiveness reported in the Pathway CH-1 study.

Our study is subject to a number of limitations. First, the Pathway CH-1 study, while randomized and sham-controlled, included only 28 patients. As a result, our trial-informed base case assumption of 67.1 % pain relief will need to be confirmed by future studies with a larger sample size. Further, the CH-1-observed distribution suggests significant patient-to-patient variation in response to stimulation. In order to test the effect of this uncertainty, we ran scenario-analyses using the lower and upper bound of the 95 % confidence interval of this parameter (50.2 %–80.5 %). Even at the lower efficacy assumption, SPG stimulation was found cost-effective, with an ICER of €18,846 per QALY gained—well below commonly acknowledged willingness-to-pay thresholds. At the higher assumption, SPG stimulation was the dominating strategy, and was associated with overall savings of €3,262. Similar findings hold for the secondary effectiveness parameter, frequency response.

Second, our analysis assumes the primary treatment effect of pain relief observed in the Pathway CH-1 study is maintained over the full time horizon of the analysis, while the secondary treatment effect of frequency reduction is gradually declining. Even though follow-up data through 24 months are available and there is no evidence or clinical rationale that would support a contrary assumption, further follow-up data will be desirable to confirm our assumptions. The same holds for the assumed longevity of the device. Our scenarios of lower and higher treatment effectiveness—albeit constant—provide insight into the potential effects of gradual changes in primary pain relief over time, and of more or less pronounced reductions in frequency response.

Third, our assumptions about medication use in the control cohort were based on a prior publication reporting on drug usage in a cohort of cCH patients treated in a tertiary headache center. While these patients share mostly comparable characteristics with the Pathway CH-1 cohort, and more broadly with patients eligible for SPG stimulation, some variation might exist in actual drug usage. To account for this uncertainty, we considered two scenarios in sensitivity analyses that assumed drug utilization increased or reduced by 25 % from baseline, respectively. Further, our model assumed that attacks successfully treated with SPG stimulation would not require any attack-aborting medication, and that patients not successfully treated would revert to the standard regimen of attack-aborting drugs. While this assumption is in line with the Pathway CH-1 study protocol, individual patient utilization might differ.

Fourth, the control cohort used in our model was defined as sharing the same characteristics as the SPG stimulation cohort at baseline, both in terms of attack frequency and health-related quality of life, and maintaining these parameters constantly over the horizon of the analysis. While this assumption seems reasonable, some fluctuations in the patients’ severity of cCH might occur, leading to higher or lower costs and quality of life. However, these potential fluctuations could be expected to occur in both the simulated control cohort and the SPG stimulation cohort, reducing the impact of any such fluctuations on the results of this analysis.

Fifth, our analysis does not consider the potential beneficial health impact of reductions in drug utilization for patients receiving SPG stimulation. Reducing the use of triptans and other attack-ending medications by more than 50 % might lead to fewer drug-related side effects. Including these beneficial effects would have made the health-economic profile of SPG stimulation more favorable.

Sixth, our economic analysis is limited to direct medical cost, that is, costs directly related to medical treatments. The preventative effects, reduction in necessary medication use, and improvement in health-related quality of life demonstrated in Pathway CH-1, however, might lead to reduced absenteeism, increased productivity, and other societal benefits not accounted for in our analysis. Including these potential benefits – again – would have increased the overall savings associated with SPG stimulation and would have further improved the favorable health-economic profile of this therapy choice.

Finally, intangible benefits such as potential therapy-related reductions in suicidal ideations and improved family/social interactions are not considered in this analysis.

## Conclusions

In summary, our findings suggest that SPG stimulation is a cost-effective treatment strategy for chronic cluster headache patients in the context of the German healthcare system. Adopting this strategy may lead to clinically relevant increases in health-related quality of life, and to overall cost savings for the healthcare system at timeframes longer than 5 years. Further clinical data are needed to confirm these model-based findings.

## Abbreviations

ATI: Autonomic Technologies, Inc.
cCH: Chronic cluster headache
CH: Cluster headache
CT: Computed tomography
DVT: Digital volume tomography
ICER: Incremental cost-effectiveness ratio
QALY: Quality-adjusted life year
SF-36: Short-form 36v2
SHI: German statutory health insurance
SPG: Sphenopalatine ganglion
